# Supplementary material for: A mast cell-ILC2-Th9 pathway promotes lung inflammation in cystic fibrosis
Source: Nat Commun. 2017 Jan 16;8:14017. doi: 10.1038/ncomms14017 (PMC5241810; doi:10.1038/ncomms14017)
Supplement: Supplementary Information — Supplementary Figures 1-8, Supplementary Tables 1-6 and Supplementary References. [file ncomms14017-s1.pdf]

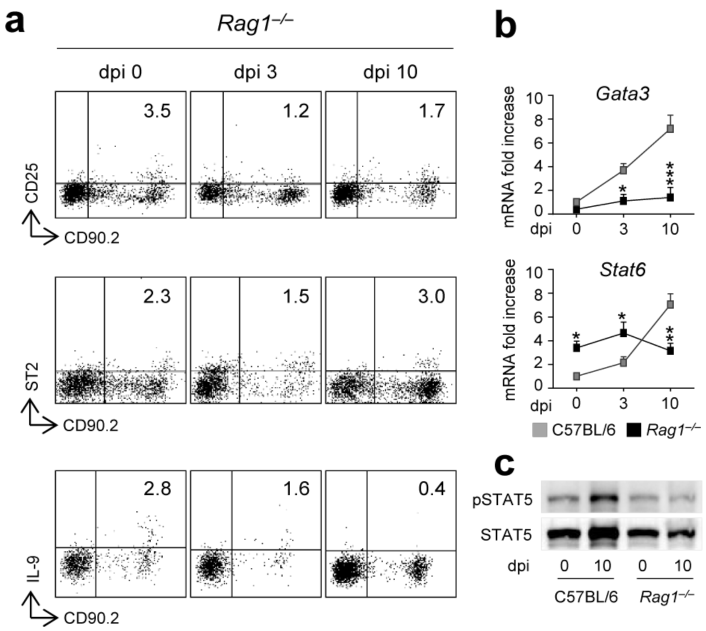

Supplementary Figure 1 ILC2 expression in *Rag1*$^{-/-}$ mice. (**a**) Detection of CD25^{+} and ST2^{+} and IL-9^{+} lung ILC2 by flow cytometry (numbers refer to percentages of positive cells), (**b**) transcription factors (RT-PCR on total lung cells) and (**c**) STAT5 and phospho-STAT5 lung expression by immunoblotting at various days post infection (dpi) in C57BL/6 and *Rag1*$^{-/-}$ mice (six per group) infected intranasally with live *A. fumigatus* conidia. Data are representative (cytofluorimetry) or pooled from two experiments. *P< 0.05, **P< 0.01, *Rag1*$^{-/-}$ *vs* C57BL/6 mice, Two-way ANOVA, Bonferroni post test.

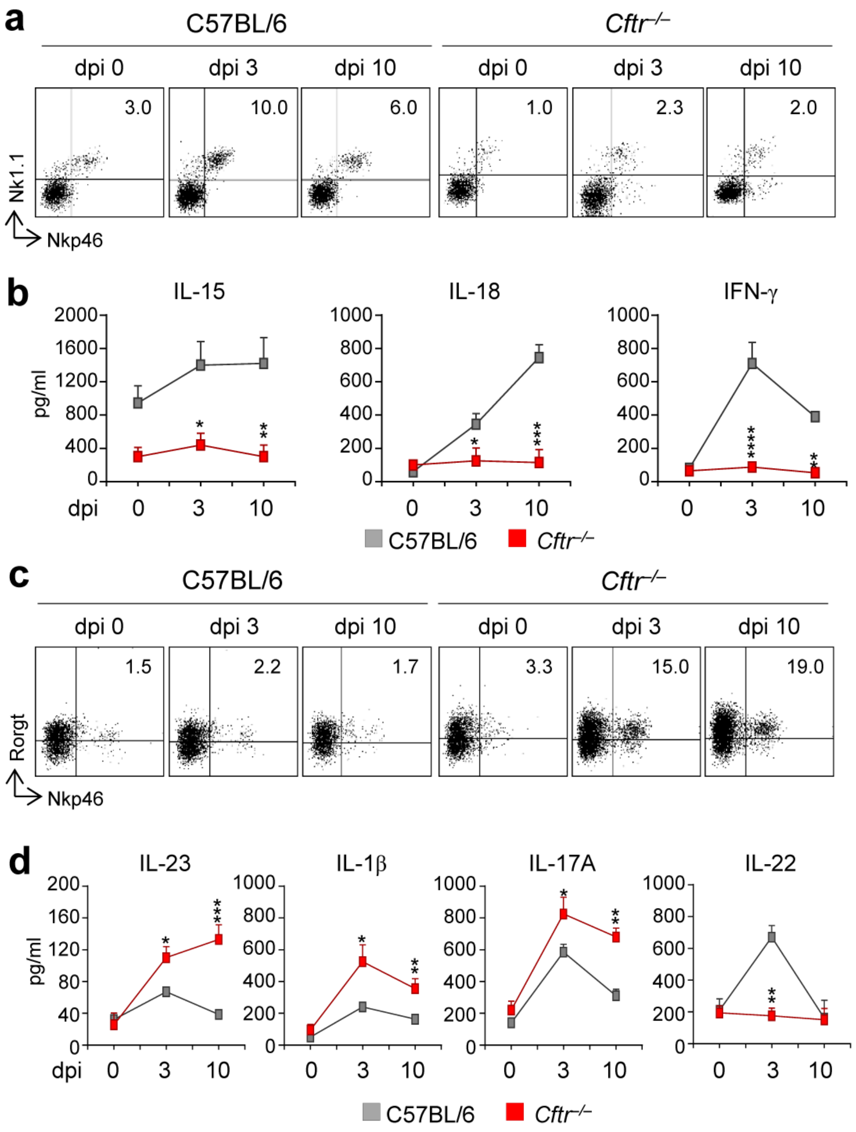

Supplementary Figure 2 ILC1 and ILC3 expansion of *Cftr*$^{-/-}$ mice during *A. fumigatus* infection. Mice (six per group) were intranasally infected with live *A. fumigatus* conidia and evaluated at different days post infection (dpi) for (**a**) cytofluorimetric analysis and relative percentage of Nk1.1^{+}Nkp46^{+} lung ILC1 and (**b**) ILC1 activating and effector cytokine production; (**c**) cytofluorimetric analysis and relative percentage of Rorgt^{+}Nkp46^{+} lung ILC3 and (**d**) ILC3 activating and effector cytokine production. Numbers in FACS plots refer to percentages of positive cells. Data are mean values ± SD, ELISA for cytokine determination was done on lung homogenates. Data are representative (cytofluorimetry) or pooled from three experiments. *P< 0.05, **P< 0.01, ***P< 0.001, ****P< 0.0001, *Cftr*$^{-/-}$ *vs* C57BL/6 mice, Two-way ANOVA, Bonferroni post test..

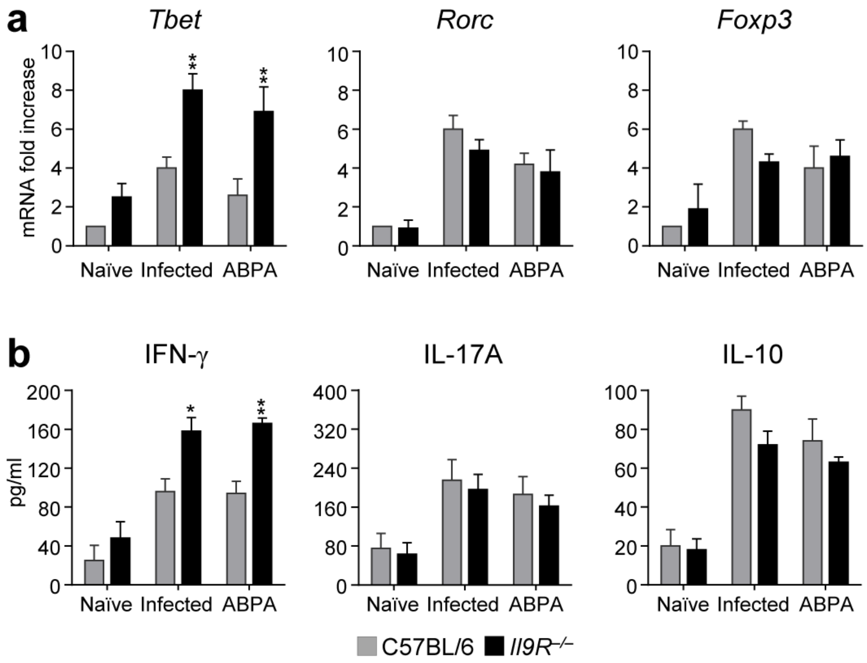

Supplementary Figure 3 Increased Th1 activation in *Il9R*$^{-/-}$ mice during invasive and allergic aspergillosis. C57BL/6 and *Il9R*$^{-/-}$ mice (six per group) were intranasally infected with live *A. fumigatus* conidia or subjected to allergic bronchopulmonary aspergillosis (ABPA) and assessed for (**a**) Th-cell specific transcripts (RT-PCR on total lung cells) and (**b**) cytokine production (mean values ± SEM, ELISA on lung homogenates). *P< 0.05, **P< 0.01, *Il9R*$^{-/-}$ *vs* C57BL/6 mice. Naïve, uninfected mice. Data represent pooled results from three experiments, Two-way ANOVA, Bonferroni post test. *Tbet* = T box expressed in T cells; *Rorc* = retinoic acid receptor–related orphan receptor C; *Foxp3* = forkhead box protein P3.

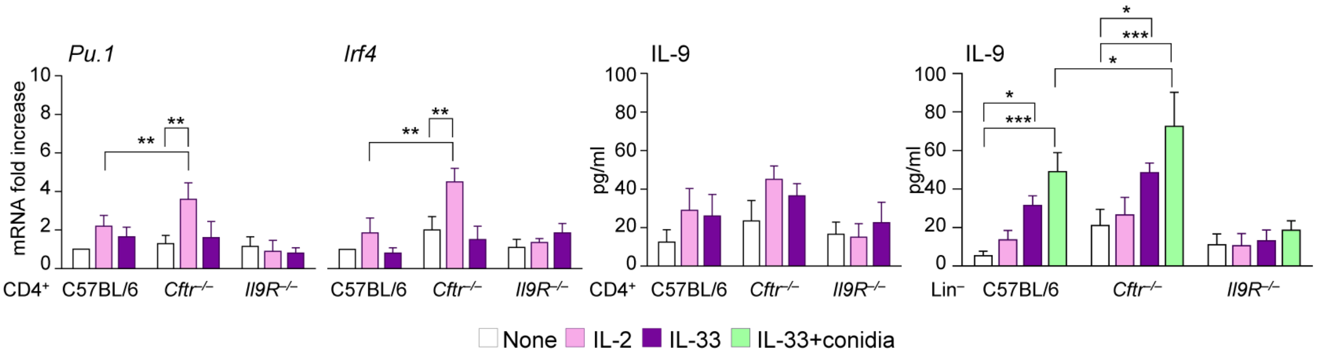

Supplementary Figure 4 Th-cell specific transcript (RT-PCR) and IL-9 production (mean values ± SD, ELISA on culture supernatants) on lung $CD4^+$ T cells and lung lineage negative (Lin^-) cells from naïve mice cultivated in vitro for 3 days with IL-2 or IL-33, in the presence of *A. fumigatus* conidia. **P< 0.01, *Cftr*$^{-/-}$ and/or *Il9R*$^{-/-}$ *vs* C57BL/6 mice. None, unstimulated cells (data represent pooled results from three experiments, Two-way ANOVA, Bonferroni post test). *Pu.1* = purine-rich box 1; *Irf4* = interferon regulatory factor 4.

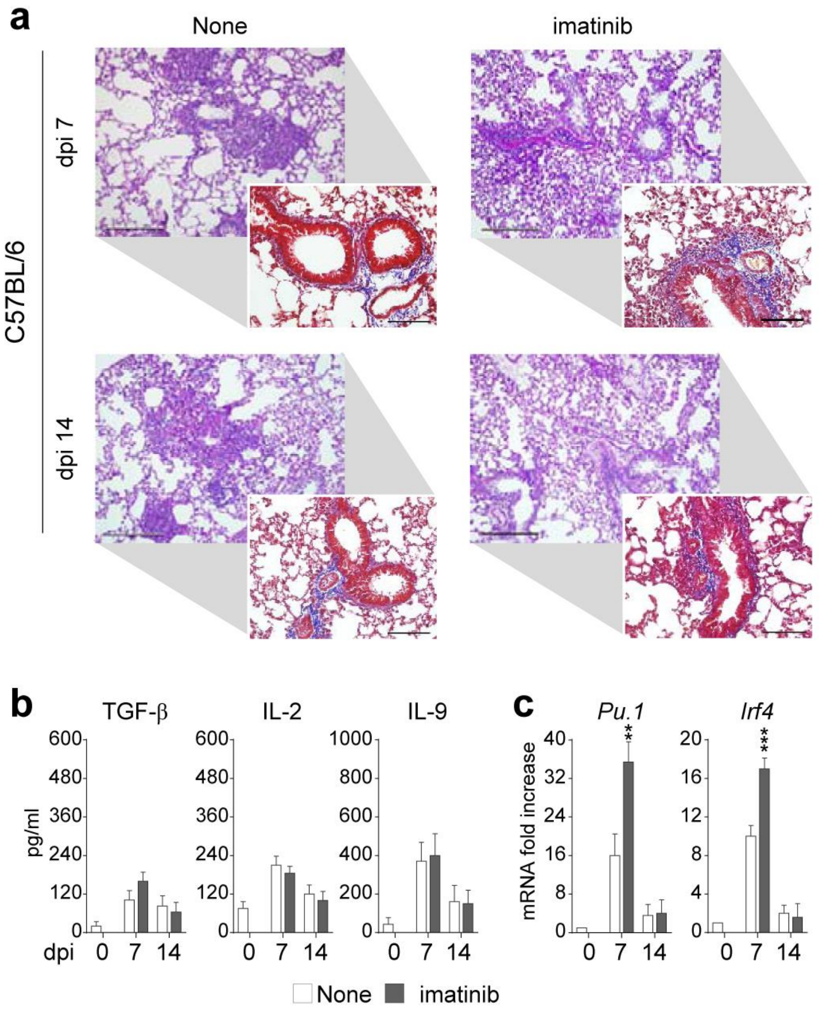

Supplementary Figure 5 Treatment of C57BL/6 mice with imatinib. (**a**) Lung histology (periodic acid–Schiff and Masson's trichrome staining, in the insets); (**b**) cytokine production and (**c**) Th9-cell specific transcripts expression in C57BL/6 mice infected intranasally with live *A. fumigatus* conidia and treated with imatinib intraperitoneally for a week. Photographs were taken with a high-resolution microscope (Olympus DP71) equipped with a ×40 objective and a ×100 objective (in the inset). Results are mean values ± SD, ELISA was done on lung homogenates for cytokines and RT-PCR on total lung cells. **$P < 0.01$, ***$P < 0.001$, imatinib-treated vs untreated (None) mice (data represent pooled results or representative images from three experiments, Two-way ANOVA, Bonferroni post test). *Pu.1* = purine-rich box 1; *Irf4* = interferon regulatory factor 4.

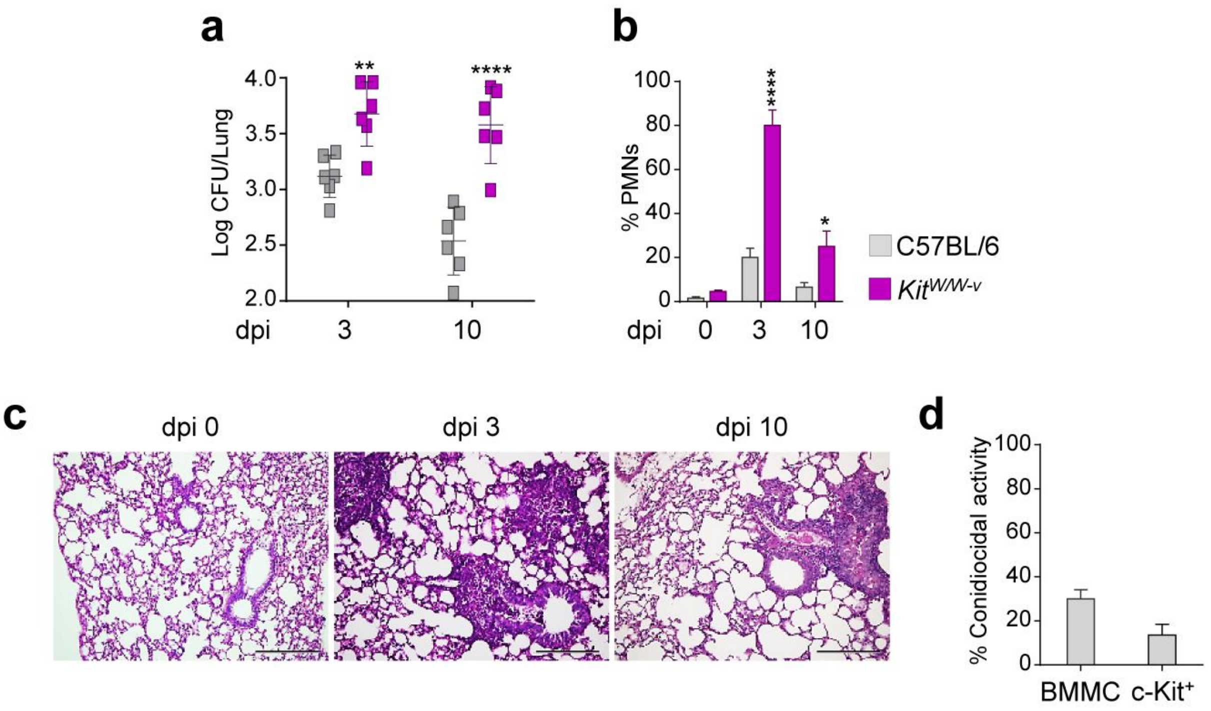

Supplementary Figure 6 Susceptibility of MC-deficient C57BL6-*Kit*$^{W/W-v}$ to aspergillosis. C57BL/6 and MC-deficient C57BL6-*Kit*$^{W/W-v}$ mice were intranasally infected with *A. fumigatus* and evaluated at the indicated times for (**a**) lung fungal burden ($\log_{10}$ cfu, mean ± SD), (**b**) polymorphonuclear cells (PMNs) recruitment on bronchoalveolar lavage fluid, (**c**) lung histology (periodic acid–Schiff) and (**d**) conidiocidal activity of bone marrow-cultured mast cells (BMMC) or c-Kit^{+} cells magnetically isolated from lung of uninfected C57BL/6 mice. Photographs were taken with a high-resolution microscope (Olympus DP71) equipped with a ×20 objective, scale bar, 200 µm. The in vivo groups consisted of six mice/group and assays were done at the indicated days after the infection (dpi). Data are representative (histology) or pooled from three experiments, Two-way ANOVA, Bonferroni post test or Two-tailed Student's T test. **P< 0.01, ****P< 0.0001, C57BL/6-*Kit*$^{W/W-v}$ *vs* C57BL/6 mice.

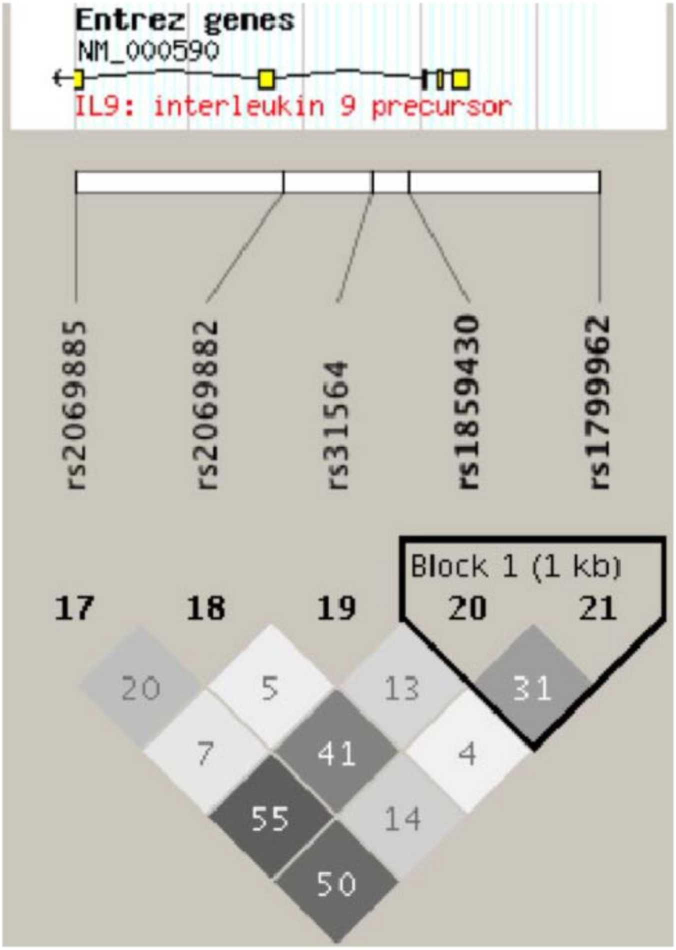

Supplementary Figure 7 Linkage Disequilibrium plot for IL9 SNPs. LD is expressed in r^2.

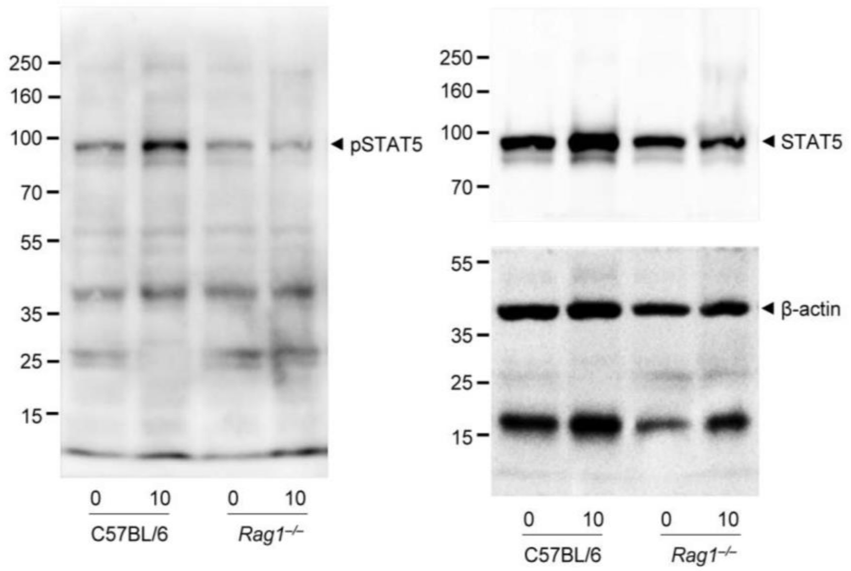

Supplementary Figure 8 Full uncut gel.

Supplementary Table 1. Demographic and clinical characteristics of the cystic fibrosis sample.

		N	Mean ± S.D.	Range
Age in years:		N = 287	15.8 ± 11.4	0.1 - 48
Age at diagnosis in months:		N = 268	30.3 ± 67.0	0 - 408
FEV1, % predicted:		N = 210	84.4 ± 26.6	21.6 – 134.1
FVC, % predicted:		N = 210	90.9 ± 21.6	29.3 – 131
BMI:		N = 217	20.2 ± 3.7	13 – 41.8
Total IgE, U/ml:		N = 237	131.7 ± 248.2	1 - 2117
		**N**	**Percent**	
Sex:	*Male*	176	50.7%	
	Female	171	49.3%	
	M/F ratio	1:1		
CFTR mutation:	*ΔF508 homozygous*	92	27.2%	
	ΔF508 heterozygous	151	44.7%	
	Other	95	28.1%	
Infection status:	*S. Aureus*	175	50.3%	
	Pseudomonas	157	46.6%	
	Aspergillus	100	30.5%	

FEV1 - forced expiratory volume in the first second; FVC - forced vital capacity; BMI - body mass index; *CFTR* - cystic fibrosis transmembrane conductance regulator. Continuous variables are expressed as mean ± SD.

Supplementary Table 2 Genotyped *IL9* SNPs.

SNP ID	Position (GRCh37/hg19)	HWE P-value	MAF	Alleles
rs2069885	Chr5:135228165	0.776	0.129	C:T
rs2069882	Chr5:135229633	0.713	0.241	A:G
rs31564	Chr5:135230253	0.433	0.354	A:C
rs1859430	Chr5:135230513	1.0	0.193	C:T
rs1799962	Chr5:135231856	0.447	0.075	A:G

Minor Allele Frequency (MAF), Hardy Weinberg Equilibrium (HWE), Genotyping rate (% Geno).

Supplementary Table 3 Single SNP analysis.

**Additive**		**Males**				**Females**			
**SNP**	**Allele**	**N**	**β**	**SE**	**P**	**N**	**β**	**SE**	**P**
rs2069885	T	115	-0.468	0.276	0.093	**118**	**0.605**	**0.296**	**0.047**
rs2069882	G	94	-0.394	0.243	0.108	94	-0.057	0.255	0.825
rs31564	C	94	-0.293	0.219	0.183	98	-0.226	0.246	0.360
rs1859430	T	94	-0.255	0.254	0.318	97	0.453	0.279	0.108
rs1799962	G	98	-0.313	0.346	0.367	98	0.536	0.358	0.137
**Dominant**		**Males**				**Females**			
**SNP**	**Allele**	**N**	**β**	**SE**	**P**	**N**	**β**	**SE**	**P**
rs2069885	T	**115**	**-0.666**	**0.310**	**0.034**	**118**	**0.624**	**0.305**	**0.043**
rs2069882	G	94	-0.480	0.292	0.103	94	-0.223	0.304	0.466
rs31564	C	94	-0.509	0.297	0.090	98	-0.356	0.301	0.240
rs1859430	T	94	-0.255	0.305	0.405	97	0.482	0.306	0.118
rs1799962	G	98	-0.458	0.420	0.278	98	0.439	0.393	0.267

Genetic association testing was carried out considering additive and dominant models by linear regression implemented in Plink v1.07[1], adjusting for age at sampling. Significant results are highlighted in bold.

Supplementary Table 4 Haplotype Analysis.

	IL9 SNPs					Males			Females		
**Haplotype**	rs2069885	rs2069882	rs31564	rs1859430	rs1799962	**(%)**	**β**	**P-value**	**(%)**	**β**	**P-value**
H1	C	A	A	C	A	43.2%	0.567	0.022	36.7%	-0.038	0.866
H2	C	A	***C***	C	A	30.2%	-0.157	0.532	32.5%	-0.313	0.221
H3	C	***G***	A	***T***	A	4.3%	0.577	0.309	6.9%	-0.325	0.434
H4	***T***	***G***	A	***T***	***G***	5.6%	-0.587	0.109	6.2%	0.195	0.680
H5	C	***G***	A	C	A	4.0%	0.233	0.668	5.8%	-0.546	0.216
H6	***T***	***G***	A	***T***	A	3.4%	-0.741	0.202	4.8%	1.160	0.131
H7	C	***G***	***C***	*C*	A	5.2%	-0.409	0.350	2.0%	0.363	0.671
H8	C	A	A	***T***	A	1.9%	-0.238	0.718	---	---	---
H9	***T***	A	A	***T***	***G***	---	---	---	1.9%	0.970	0.107
H10	***T***	A	A	***T***	A	---	---	---	1.6%	1.120	0.135
						Omnibus P=0.221			Omnibus P= 0.132		

Haplotype association testing was carried out by general linear model implemented in Plink v1.07[1], adjusting for age at sampling. Minor alleles at each SNP are reported in italics bold. An omnibus association test was used to test all haplotypes combined for association with IgE levels.

Supplementary Table 5 Real-time PCR primers used in this study.

Gene name	Primer sequence
**Murine Primers**	
Tbet	Forward, 5′- GGACGATCATCTGGGTCACATTGT -3′
	Reverse, 5′- GCCAGGGAACCGCTTATATG -3′
Gata3	Forward, 5′- TCTGGAGGAGGAACGCTAATG -3′
	Reverse, 5′- GGCTGGAGTGGCTGAAGG -3′
Rora	Forward, 5′- GGTCGGATGTCCAAGAAGCAGAG -3′
	Reverse, 5′- GATGTTGTAGGTGGGCGTCAGC -3′
Rorc	Forward, 5′- ACAACAGCAGCAAGTGATGG -3′
	Reverse, 5′- CCTGGATTTATCCCTGCTGA -3′
Foxp3	Forward, 5′- CCCAGGAAAGACAGCAACCTTTT -3′
	Reverse, 5′- TTCTCACAACCAGGCCACTTG -3′
Stat6	Forward, 5′- CACATTTTGGCAGTGGTTTG -3′
	Reverse, 5′- CTGGCTCATTGAGGAGAAGG -3′
Il9	Forward, 5′- TGACCAGCTGCTTGTGTCTC -3′
	Reverse, 5′- GTGGCATTGGTCAGCTGTAA -3′
Pu.1	Forward, 5′- GGTCCTAACCCCTCCACCTA -3′
	Reverse, 5′- TCTGGCTGGTGAAGTCCTCT -3′
Irf4	Forward, 5′- GACCAGTCACACCCAGAAATCC -3′
	Reverse, 5′- TGGGGCACAAGCATAAAAGGTT -3′
Tph1	Forward, 5′- GAACAGTTGAATGACATCTT -3′
	Reverse, 5′- AAGGGACAGTCTCCATAA -3′
Mcpt1	Forward, 5′- TCGAAAAACAAATCATTCACAAA -3′
	Reverse, 5′- GACCAGGCAAGGGAATTACA -3′
Mcpt6	Forward, 5′- TTCTGCGGAGGTTCTCTCAT -3′
	Reverse, 5′- TACTGCTCACGAAGCTGCAC -3′
**Human Primers**	
**Gene name**	**Primer sequence**
IL9	Forward, 5′- CCAATACCACCATGCAAACA -3′
	Reverse, 5′- CATGGCTGTTCACAGGAAAA -3′
IL9R	Forward, 5′- CGTGCCCTCTCCAGCGATGTTCT -3′
	Reverse, 5′- GACGCGCTGGGCCACAAGTG -3′

Supplementary Table 6 Power calculation for the association of IL9 rs2069885 with total IgE level in our sample study.

R^2	Power
0.010	0.339
0.015	0.473
0.020	0.590
0.025	0.688
0.030	0.766
0.035	0.828
0.040	0.875

SUPPLEMENTARY REFERENCES

1 Purcell, S. *et al*. PLINK: a tool set for whole-genome association and population-based linkage analyses. *American journal of human genetics* **81**, 559-575, (2007).
